# An ontological turn in the sociology of personal life: Tracing facet methodology’s connective ontology

**DOI:** 10.1177/00380261231212099

**Published:** 2023-11-29

**Authors:** James Rupert Fletcher

**Affiliations:** Department of Sociology, University of Manchester, UK

**Keywords:** ecology, humanism, new-materialism, sensation, vitalism

## Abstract

The sociology of personal life (SPL) has been largely untouched by sociology’s ontological turn. A few scholars have attempted to retrofit SPL and new-materialist ideas, but these limited attempts have overlooked the potential for SPL to furnish its own definitive ontological contributions in dialogue with the wider turn. In response, I offer an SPL-centred ontology: the ‘connective ontology’ of facet methodology. Introduced in 2011, facet methodology was originally supported by ‘connective ontology’, briefly outlined in a preliminary text, but as facet methodology has gained popularity, connective ontology has not been explicitly discussed. I argue that, while never mentioned outright, Jennifer Mason’s 2018 *Affinities* is about connective ontology and therefore offers an extensive, albeit tantalisingly implicit, ontological thesis, which I attempt to develop. In my interpretation, the world is made up of fluctuating layerings of vitally animated connection units. This connective ontology resonates with contemporary new-materialist sensibilities, but is *from* and *of* SPL. It also speaks to broader theoretical traditions, offering provocations for ontologically-minded, live, post-humanist, vitalist and biosocial sociologies, especially regarding longstanding critiques of apoliticism.

In this paper, I cross-fertilise the sociology of personal life (SPL) and the ontological turn, two popular but relatively disconnected sociological fields. I do so by explicating ‘connective ontology’, a flat ontology that emerged from SPL independently of the ontological turn, but which nonetheless resonates with it. In 2011, connective ontology was briefly outlined as a basis for facet methodology, but as facet methodology has spread, connective ontology has not been discussed explicitly. I argue that Jennifer Mason’s (2018) *Affinities* offers an extensive, albeit implicit (it is never directly mentioned), engagement with connective ontology, and I attempt to build on that ontology in reference to resonant scholarships. My result – a social ontology of dynamically emergent vitally animated connections – intersects SPL, the ontological turn and other contemporary sociological concerns, reimagining the forms and impacts of social relations as themselves being fundamentally ontological matters.

Connective ontology has several noteworthy sociological implications. It provides SPL with an explicit ontological footing that can be indeterminate in the subfield, linking SPL into sociologies from which it is often isolated, e.g. Latour, Barad and Simmel. It respects the ontological turn’s characteristic monism but pragmatically maintains humanistic sensibilities as a basis for doing transformative sociology. It thereby resonates with recent calls for live sociologies that cultivate radical ethico-epistemological methodologies in response to real-world phenomena. It also offers tools for SPL engagements with uncertainties regarding more-than-human theorisation, participation and representation, as well as speaking to the vitalist turn by attending to the everyday vitalities of personal life. In sum, I show that connective ontology can enrich SPL and connect it into several traditionally inconsonant sociological territories, to mutual benefit.

## Introduction: The personal is ontological

Since the mid-2000s, sociology’s ‘ontological turn’ has challenged earlier materialist and constructivist commitments to realist and non-realist ontologies respectively, instead advocating more unorthodox engagements with the nature of reality (Pickering, 2017). Scholars have drawn inspiration from an eclectic combination of quantum physics and indigenous philosophy to explicate ontologies that are variably relational, processual and multiple (Gullion, 2018). The resulting post-humanist and new-materialist ‘flat’ or ‘monist’ ontologies that have characterised this turn have popularised concepts such as ‘intra-action’, ‘entanglement’ and ‘assemblage’ across sociology (Fox & Alldred, 2016; Giraud, 2019).

However, despite significant influence elsewhere, the ontological turn has been relatively unremarked upon in SPL, a sub-discipline that is intuitively conducive to relational views of existence. Indeed, Roseneil and Ketokivi (2016) characterise SPL as deeply attached to the concept of relationality, but without theorising its ontological status, or indeed any substantive ontology. Their proposed solution is for SPL to engage with the longstanding ontological commitments of American pragmatism, figurational sociology and psychoanalysis, again overlooking the ontological turn. In contrast, Mauthner (2021) has championed Barad’s (2007) agential-realist onto-epistemology (discussed below) as a basis for SPL, centring on intra-action. Taking a different approach, Chiew and Barnwell (2019) have used twin intimacies to reflect traditional SPL subject matter back onto a loosely conceived relational ontology. They note that twins have long unsettled nature–nurture dualisms, manifesting a mysterious entanglement of the material and immaterial.

The respective works of Mauthner, Chiew and Barnwell are dedicated attempts to nurture an SPL ontological turn in line with sociology more generally, but each has sought to transpose the wider ontological turn onto SPL, and neither has subsequently been taken up in any substantive manner. In this paper, I propose a different – indeed, the opposite – solution. I argue that ontologies aligned with contemporary new-materialist sensibilities can be explicitly demarcated from within SPL itself. To do this, I attempt to explicate and extend the ‘connective’ ontology that originally underpinned facet methodology, but which has not been explicitly discussed during the past decade.

I begin by summarising facet methodology and its sociological implications. I then outline connective ontology as far as it has been explicitly defined and developed to date (that is, not much). In response, I argue that, while never mentioned explicitly, a sophisticated account of connective ontology is implicit throughout Mason’s (2018) monograph *Affinities*, which I briefly outline. I then trace (i.e. pursuing and emulating, but inevitably transforming) a connective ontology through *Affinities* vis-a-vis three lines of critique: (1) conceptualisations of connection as a basic unit of social analyses; (2) tantalisingly incoherent commitments to ecological holism and human-centred sensations; and (3) resonances with processual vitalism. Ultimately, connective ontology is a stimulating approach to the nature of human existence and the study thereof. It offers provocations for sophisticated and innovative research, bringing SPL and the ontological turn into dialogue, and potentially speaking to several other seemingly discrepant sociologies.

## Facet methodology

In 2011, Jennifer Mason (2011) published a detailed account of facet methodology, an expansive approach to investigating the ‘multi-dimensionality of lived experience’ and capturing the vital dynamics of relationalities (p. 75). It transgresses orthodox methodological formalities and preconceptions, especially regarding traditional unilateral methodological approaches, by engaging with multifaceted phenomena in multifaceted ways. It extends the argument for mixing beyond an epistemological advocation for mixed-methods, making a more expansive ontological claim that both methodologies and epistemologies should be multifaceted because our study phenomena are themselves essentially multifaceted, with those methodologies and epistemologies themselves partly comprising our research problematics. Hence, facet methodology sits amidst 21st century appeals to a ‘live sociology’, characterised by an imaginative methodological artistry that corresponds to radical ethico-epistemological commitments (Back & Puwar, 2012).

Facet methodology begins from the premise that research fields are constituted by numerous facets. Consider a random sociological study, e.g. drug consumption in snooker halls, exotic pet ownership in student accommodation, meme use in 60+ chatrooms. Each research problematic is reasonably defined, yet each comprises different angles and approaches. It is odd, then, that we might conventionally study them by following a given method, perhaps because that method seems most applicable to the problem (or perhaps simply the easiest thing to do). We might conduct participant observation in a snooker hall, interview student pet owners or discursively analyse meme posts. Mason emphasises the tension inherent in applying a given method to a sociological problem. Instead, she suggests iteratively prioritising different lines of investigation relative to evolving intuitions, theories and research, leading to clusters of methods.

Mason (2011) uses a cut gemstone metaphor to exemplify facet methodology. Each cut face sheds, reflects, refracts and intensifies light relative to the entire object. Alone, each cut gives the observer a glimpse into the nature of the gemstone, but in combination, these cuts and the glimpses they afford offer an amalgamated perspective on the gemstone as more than the sum of its faces. Sociologically, the gemstone represents the research problem, and each facet represents a particular empirical engagement with that problem. Hence, the facet methodologist curates clusters of methods that prioritise different angles and approaches. Mini studies constitute facets, which in combination strategically illuminate the research problematic. Each facet is a methodological plane of the research problem, as each cut face is a surface of the gemstone. This means that epistemology is inseparable from ontology, and that epistemology is intrinsically indicative of ontology.

Mason offered facet methodology as a provocation for sociological debate on methodological innovation. The original paper was a call-to-arms, and facet methodology has subsequently been taken up and adapted across several sociologies. Besides SPL (e.g. Lewis et al., 2018), it has been used in family sociology (Barnwell, 2022), political science (Bruff, 2017; Cavaghan & Elomäki, 2022), media studies (Bramley, 2021; Muir, 2022), creative methods (Meckin & Balmer, 2021), medical sociology (Phoenix & Bell, 2019), migration studies (Sedmak & Medarić, 2022), digital sociology (Ogden, 2020; Wilson, 2015), the sociology of education (Bamsey, 2021) and STS (Fletcher & Birk, 2019). Throughout, facet methodology has provided a means of doing multifaceted and holistic research, holding sociological methods in dialogue with the social world (Muir, 2022).

## Elusive connective ontology

One aspect of Mason’s original outline that has almost never been explicitly written about since is ontology, an underdefined facet of facet methodology that I wish to explore as an SPL contribution to the ontological turn. As noted, facet methodology is not simply an epistemological advocation of mixed-methods. It goes further, making an ontological argument about the parallel and entwined multifaceted natures of epistemology, methodology and sociological study phenomena. Indeed, facet methodology makes several assumptions about the nature of sociological research problems – they are multifaceted, relational, dynamic and vital. Mason summarised this set of commitments as ‘connective ontology’ in her original paper, describing it as a ‘strongly anti-reductionist’ conception of the world that is inherently ‘multi-dimensional, contingent, relationally implicated and entwined’ (p. 78). This definition is the limit of her explicit ontological description.

We can slightly expand this brief characterisation of connective ontology by attending to what it is not. Mason states that the gemstone metaphor, while helpful in several respects, is unhelpful inasmuch as the gemstone is solid, inert, sharply defined and self-contained, at least intuitively, in a manner alien to sociological research problems. She explicitly demarcates these qualities as ontologically misleading, which can be interpreted as indicating something about connective ontology via contrast. If connective ontology is not solid, inert, defined or contained, then it seems reasonable to extrapolate that it is fluid, vibrant, blurry and unbounded.

We know little more about the connective ontology underpinning facet methodology. It is the subject of a single brief section of Mason’s original paper. That brevity is understandable, because ontology was one of six defining principles in one section of a word-limited introductory article, but it is regrettable for two reasons. Firstly, connective ontology offers considerable potential for enriching an ontological turn from within SPL, and informing sociology more broadly. Second, it risks being side-lined in engagements with facet methodology, which is now proliferating across sociology. As noted, various scholars have applied facet methodology to different research problems. However, none of these engages with the connective ontological implications of facet methodology beyond cursory mentions. None of the examples cited above references connective ontology at all. This is not to say they are naïve to it – a lack of explication does not indicate a lack of awareness. Rather, these works offer no explicit ontological reflection or development.

Fleeting mentions can be found in Brownlie’s (2020) study of refugee memory. However, these are limited to single sentences that reiterate Mason’s original definition. There is also a chance echo in one paper on the phenomenology of animal life (Lestel et al., 2014). Here, ‘connective ontology’ is mentioned explicitly, in isolation from Mason’s writing, as a critique of 19th century ecological and biological taxonomy. These taxonomies abstracted animal specimens from their ecologies and thereby, the authors claim, forfeited insights into those animals and their existence, because they are essentially ecologically entwined. This is a serendipitous indication of the potential of connective ontology. However, as will become apparent below, this argument manifests much of Mason’s more implicit treatment of connective ontology.

The lack of ontological explication in subsequent facet sociologies is understandable. Mason advocated that doing facet methodology did not require rigid adherence to connective ontology, and instead encouraged a general alertness to the contingent entwinement that typified multidimensional existence. Indeed, the premise of the present paper – tying down and bulking up a connective ontology – is at odds with Mason’s positioning of it relative to the more central issue of doing facet methodology. However, the focus of this paper is not doing facet methodology, but rather the connective ontology that its doing gestures toward. Fortunately, despite its explicit disappearance from sociology, connective ontology remains. In the next section, I suggest that Mason’s later work offers a key resource for engaging with connective ontology and briefly characterise that work.

## *Affinities*: An untapped reservoir

My contention is that a connective ontology can be traced through Mason’s 2018 monograph *Affinities*, which provides considerable insight into, and indeed is arguably about, connective ontology, albeit without ever explicitly mentioning it. This stark inexplicitness could be read as exemplifying Roseneil and Ketokivi’s (2016) aforementioned criticism of SPL as ontologically agnostic. However, the text might be better read as offering a response to that criticism, because it implicitly contains an ontology that is *from* and *of* SPL, and that can be multiply interpreted and developed for more explicit sociological treatments (i.e. this paper).

*Affinities* (Mason, 2018) argues that life is characterised by potent connections that traditional sociologies are unalert to or unable/unwilling to engage with (pp. 200–202). These connections, or affinities (used synonymously), are vital energies that comprise the locus of everyday experience. They come in innumerable forms: recognising resemblances in long-lost family or being transported across time and place by a smell. Affinities emanate in such happenings, both mundane and ethereal, introducing a mystical vitality into SPL. Mason (p. 2) emphasises that affinities extend beyond us and the things that are connected. Hence, *Affinities* is not about emotions or psychological impulses of attachment, nor does it attend to the things connected. Connections themselves are the fundamental concern.

*Affinities* develops four themes: sensation, kinship, ecology and time. Beginning with ‘sensations’, Mason (pp. 7–10) highlights the ability for a song or smell to suddenly overwhelm and transport us to a distant experience. Rather than intrinsic human perceptions of extrinsic stimuli, sensations here are understood to flow through and emanate from encounters, vitalising affinities. Sensations encompass traditional classifications – e.g. sight – but also unorthodox forms – e.g. ambiance, memory. Mason (pp. 59–62) uses ‘kinship’ to explore the potency and ineffability of affinities: ‘From the adoptee who yearns for relatives she resembles, to the young man who neighbours say has inherited his criminal father’s bad blood, to yourself when you realise you have your sister’s laugh’ (p. 60).

Mason (pp. 123–125) uses ‘ecology’ to explore world-constituting layerings of affinities. She depicts ecologies as constantly evolving layerings of connection, animated by the living of lives, including animals, technologies, climates, etc. Ecologies are not additive assemblages of discrete things. They are indivisible nexuses of vital intra-animations. Mason (pp. 188–190) ties together sensation, kinship and ecology via ‘time’. As an all-pervasive medium within which happenings happen, time can become everything and nothing, but such abstract omnipresence underplays time’s connective potency. My affinities with a patch of grass on my childhood estate are not timeless. They connect me to a time-bounded ecology of the early 2000s, even to a climatically-time-bounded ecology of summers, sunburn, hay-fever and wasps. The temporality enhances the potency.

Given Mason’s creation of connective ontology, it is unsurprising that *Affinities* is grounded in concordant commitments. Tantalisingly, *Affinities* is the most thorough and sophisticated realisation of connective ontology, yet connective ontology itself is never mentioned. I am not suggesting that Mason is unaware of her ontological commitments. Given her earlier writing, this is evidently untrue. While explicitly silent on the subject, implicitly the book can be read as a text about ontology. Hence, *Affinities* answers its own demands for sociological development, broaching an SPL-centred ontological turn. In what follows, I develop that answer by explicating a till-now implicit connective ontology, traced through *Affinities* in relation to alternate sociological scholarships.

## Connection units

I begin with basic ontological building blocks: connections. The concept of connections evidently carries great weight in the connective ontology underpinning *Affinities*. Mason (p. 200) is clear that traditional empirical emphasis on things – people, items, ideas, places, etc. – is not how she understands affinities. *Affinities* feels most like an ontological text when it turns to the conceptualisation of things in (intra)relation to ecology. Mason claims: ‘the [dictionary] definition of organisms existing in relation to physical surroundings already assumes a certain ontology that, in fact, we may wish to question insofar as it implies that organisms are fixed entities or that surroundings are physical or material’ (p. 123). This passage in particular represents the influence of Tim Ingold’s work on connective ontology regarding ecology.

Ingold’s (2000) *The Perception of the Environment* argued for a holistic ontology of human life as active, relational and ecological. Traditionally, much social theory and corresponding empirical work has disaggregated human life into biophysical and sociocultural building blocks, which are brought into dialogue through a vaguely defined psychological interface, binding the two distinct types of stuff together to form a composite (Fletcher & Birk, 2022). Ingold rejected this composite ontology, and instead advocated a holistic ontology wherein things fundamentally exist in an entwined state, to the extent that indivisible entwinement was existence.

Mason (pp. 45–46) adapted Ingold’s ontology of fundamental entwinement. Her pronounced commitment to holism and anti-reductionism reiterates Ingold’s position. She rejects ‘conventional analytical and categorical separations’, which she attributes to an ‘arrogant and naïve’ humanism (p. 124). However, *Affinities* goes further toward an exclusive relational essentialism. Perhaps the most significant peculiarity of connective ontology is that the foundational unit is not the particular things, which Ingold critiqued, nor the holistic entwinement that Ingold advocated. Instead, connective ontology centres connections themselves. Mason (p. 171) uses Ingold’s holism to move beyond a focus on the component things that are connected, because the deconstruction of ecologies into component pieces – which she criticises assemblage scholarship for – is a misleading intellectual imposition onto a world that does not exist in such a fragmentary state.

Entanglement and assemblage have been cornerstones of the ontological turn under the influence of new-materialism (Fox & Alldred, 2016; Giraud, 2019). ‘Entanglement’ has been taken up across much sociology to variably denote the difficulty, futility and/or even impossibility of disentangling people from other things and beings (Giraud, 2019). Such notions of entanglement as a fundamental qualification of living have reinvigorated longstanding feminist commitments to relationality as an ontological concern, supporting influential theoretical scholarship (e.g. Barad, 2007; Haraway, 2008). Similarly, the concept of ‘assemblages’ as temporary relational networks with forms of agency was popularised following Latour’s (2005) work on association, again arguing for new sociological engagements with material and ontology. Here, assemblages are made up of human and non-human actants existing in relation, the relating of which is agential because relations have their own capacity to affect.

Diverging from mainstream new-materialist commitments to entanglement and assemblage, Mason (pp. 2, 183–184) articulates connections as a meaningfully distinct and more fundamental concern from that which is connected (though connections should not and cannot be isolated from those things). One might interpret this as an intensification of Latourian assemblage, whereby the assemblage is forfeit and only the agency remains. She conjures a world in which the things that we conceive of as things are not connected so much as they are made up of connections that run through them and extend into, and partly make up, other things. It becomes more meaningful to say that things are connected insofar as those connected things are themselves made up of connections, and so on. Connections all the way down and all the way out. Bluntly, we might say that the base ontological unit in *Affinities* is the connection (or the affinity).

Taken as such, this basic connective ontology could inform explicit ontological theses in SPL. For instance, the conceptualisation of the ‘personal’ is a key issue in SPL, but where it is unpacked it is often considered theoretically or linguistically vis-a-vis empirical examples, rather than ontologically (Morgan, 2019). Likewise, ‘relationships’ are core SPL fodder and are subject to considerable conceptual interrogation, sometimes with an ontological bent, but typically under-explicated (Roseneil & Ketokivi, 2016). More often, relationships are depicted as patterns of social action reflecting social forces, e.g. norms, discourses, again focusing on the hidden structuring of quotidian human happenings (Nordqvist, 2019). Ontologically, these discussions implicitly adopt a pseudo-cognitivism of beings doing meaning within structure–agency nexuses (Fletcher, 2023). Connective ontology could invigorate such scholarships by encouraging dedicated exposition of ontology in SPL research and simultaneously providing the basis for methodological decision-making and links to wider sociologies.

The centrality of connection to connective ontology raises the question of what these connections are. We must remember, when defining, capturing or identifying underlying connections, that connective ontology rejects typification outright, of both the things that are connected and the connections themselves. Indeed, Mason calls out typification as being ontologically alien to the world described in *Affinities*: ‘Scientific classification . . . take[s] shape in an altogether different register, expressing different ontologies’ (p. 180). Therefore, a sociology guided by connective ontology should avoid any impulse toward typifying connections into any hard schema. Connections do not exist in a categorical form. To subject them to such an intellectual imposition would carry us further from their nature and obscure rather than illuminate.

Building on this rejection of typification, Mason repeatedly describes ‘the ineffable . . . nature of affinities’ (p. 44), particularly when introducing kinship (pp. 59–62). However, this does not entail giving up on gaining or articulating insight, and she therefore takes steps to lessen that ineffability. She carefully clarifies that connections are not emotions as we might traditionally conceive them, wherein a person might experience some sort of psychological impulse toward a close acquaintance. Instead, affinities are defined as charges, flows, forces and energies, largely as a means of capturing their characteristic motion, vitality and generative capacities: ‘affinities are connective charges, forces and flows, rather than static relationships between fixed entities’ (p. 123). This means that affinities are not simply formulaic ties between things, as might be denoted by a straight line between two nodes on a diagram. They are fluid connections that partly owe their vitality to other connections and are generative in a manner that warrants dedicated investigation.

Repeated appeals to connections as energy resonate with the new-materialist scholarship of Barad (2007) regarding agential-realist onto-epistemology. Agential-realism is inspired, metaphorically, by quantum physics, wherein the material basic units of classical physics are replaced by probabilistic waves that exist in a state of potentiality and only materialise when intra-acting. Here, the foundational nature of moving energies, rather than inert objects, begets a worldview in which connection is uniquely and singularly formative. Nothing precedes connection besides the possibility of connection. Agential-realism has been influential in the ontological turn generally (Fox & Alldred, 2016) and is also the focus of Mauthner’s (2021) aforementioned attempt to strengthen SPL’s ontological sensibilities. Connective ontology is less grandiose than agential-realism because Mason avoids any absolute claims about connection as materially prior. However, the comparison is informative because Barad details an ontology of energy and connection that is committed to matter but dismissive of traditional materialism.

While resonances with Barad’s more overtly ontological work may shed some light on the primality of connections as generative energies, characterising connective ontology as new-materialist is a stretch given the ethereal immateriality that is reiterated throughout *Affinities*. By this, I mean that the focus is explicitly set against things and toward energy, but not in a fashionable quantum sense whereby energy constitutes matter, but rather that matter is sociologically insufficient in lieu of its connections and connectedness. There is no attempt to define this energy, but rather to richly describe its flows and functions, to evoke some sense of it in the reader. This is where the agential-realism comparison is most informative, highlighting the simultaneous grandiosity (shared with agential-realism) and mundanity (not so shared) of connective ontology, echoing the nature of affinities themselves. Hence, connective ontology is a monism that is distinctively of SPL.

Mason (p. 1) repeatedly acknowledges the ineffable ephemerality and ethereality of personal life, emphasising its mysticalness, while also attempting to capture, comprehend and convey flashes of it. Of course, it cannot be captured, comprehended or conveyed, but the Sisyphean task of sociology is to forever attempt to do so in imaginative ways. This onto-epistemological radicalness is rendered more stimulating by its parochial relatability to human life. Used well, connective ontology allows the sociologist to generate those ‘oh yeah’ moments of pleasing insight that change how we view the taken-for-granted. We can all appreciate instances of intangible kinship, sensory time-travel, etc., but rarely reflect on their magic as social things in their own right, as the stuff of human life. Hence, prevailing new-materialist flat ontologies are replaced by a partially human-animated social ontology. Again, there is a rejection of more grandiose ontological claims, in favour of social life as a swell of quotidian happenings. This semi-humanism is a prickly aspect of connective ontology because it feels wrought with tensions regarding the human/post-human. These tensions speak to contemporary sociological debates and warrant further reflection.

## (Almost) more-than-human

Considering humanism, it is important to clarify that there is no semiotics-obsessed constructionism in *Affinities*. Expansive poetic metaphors are used as an epistemic device to shed light on affinities, but the basic reality of potent connections is explicitly demarcated as being ontologically independent of people, their minds and their languages. Again, there are shades of new-materialism here, whereby units (in this case connections) exist autonomously, but that existence is manifest in energetic enlivenings relative to living, so it is ontologically indebted to, if not contingent on, perception, albeit loosely conceived. This equivocation is emblematic of the uneasy back-and-forth between connections as independently existing and connections as animated by life. Things do not matter so much as the connections between them – this is the basic statement of *Affinities*. Nonetheless, the connections that are discussed are mostly a particular type of connection, involving at least one human in some capacity. This is a substantial tension in connective ontology, oscillating between humanist and post-humanist sensibilities in a manner that never allows the relationship to settle.

The focus on sensations intensifies this observation, so that the tension between connective ontology and (post-)humanism feels provocatively incomplete in *Affinities*. The sensation-centrism echoes the wider 21st century reclamation of the senses as both sociological method and subject (Back & Puwar, 2012), but in *Affinities* sensations are far beyond orthodox notions of seeing, hearing, etc. Sensations are extrinsic, emanating in connections, transgressing orthodox neurocognitive (p. 16) or phenomenological (p. 148) conceptualisations. Mason specifies that sensations are not reducible to neuronal stimulus–response mechanisms, nor to interpretive meaning-making. She argues that both views misleadingly centre the person and overlook how sensations emanate via environment–person relations, being attributable to neither in isolation (p. 45). However, a vocabulary of sensation and the senses inevitably draws us toward considerations of sense, corporeal senses and the subjective sensing of sensations.

Sensation is difficult to abstract from the sensing human subject, particularly considering that nothing non-human (perhaps excepting AI) is likely to experience and articulate affinities. Of course, this is disputable. Who is to say that a trout’s somersault on a summer evening is not a means of realising and expressing some flow of affinities entirely alien to us? This opens up questions regarding whether connective ontology could underpin a sociology of everyday more-than-human lives, particularly given diverse sensory apparatuses – the trout’s lateral line, the bat’s echolocation, perhaps even the reactive capacities of inanimate compounds (e.g. water transitioning through different states in relation to pressure and temperature). Though as yet unrealised, connective ontology could contribute to contemporary post-humanist epistemological debates in sociology. It exemplifies Liddiard and colleagues’ (2019, p. 1473) appeal to ‘blend[] the pragmatics of humanism with posthuman possibilities’ as a means of simultaneously implementing critical theory and action, as well as Braidotti’s (2018, p. 39) call to ‘take real-life events seriously’. Real-life is core SPL fodder, and connective ontology can bring that life into firm dialogue with these post-humanist scholarships, particularly vis-a-vis sensation.

While pragmatically foregrounding humans, the focus on sensations does capture the often-involuntary ways in which affinities arise. Accordingly, there is no prerequisite agency in *Affinities*, but rather a (semi-)agential-realism whereby the connections themselves are pseudo-agentic. Consider Mason’s discussion of ineffable family resemblances as unexpectedly striking people: ‘Exploding into a connective charge between them that has the power to startle and surprise’ (pp. 86–87), ‘hit[ting] you in a flash . . . only to disappear when you . . . try to hold everything still’ (p. 117). The observer is overwhelmed by some sense of similarity that is potent, unwilled and partly indescribable. Something is happening that transcends the sensing subject or the subjects of those senses, instead flowing between them. Here, human agency is downplayed, but in other instances it can be important. Mason uses kinship to reveal the crafting of affinities, or at least attempts in this direction. For example, parents of surrogate children can express scepticism toward the importance of genetics in family resemblance, and instead emphasise the generative role of formative experiences. Such ‘practices of kinning’ might be read as a deliberative pursuit of affinities, though it is questionable whether they can be knowingly curated (p. 96).

This work on kinship as both deliberative and startling indicates that the connections of connective ontology are co-constitutive in a manner that undermines notions of agency and subject. They can be variably incidental and purposeful, simultaneously extrinsic to and running through people, and made potent through vital sensory-kinaesthetic registers. Hence, the world is an entwinement, or a transcendence, of the subjective and the objective. Hinting at this ontological characterisation vis-a-vis kinship, Mason notes: ‘One of the fascinations of resemblance is that it often seems to defy any . . . rules of regularity, including genetic ones, but also ideas of . . . social construction’ (p. 117). The boundary-dismissing influence of Ingold, via appeals to holism and vital energies, is particularly evident here.

Nonetheless, even if it de-emphasises agency, *Affinities* never abolishes a certain immediacy of the human as a sensing subject in relation to sensations. I would argue that this represents a disciplinary predilection toward humans in a book heavily grounded in SPL. It is unclear whether such a sociology can escape humanism (not that it would or should wish to). Quotidian personal life is central to *Affinities*, and is repeatedly articulated in terms of personal experience, again positioning affinities as implicitly person-contingent. This contingency draws on Merleau-Ponty’s (1945/2002) phenomenology of embodiment, whereby one’s perception is a dynamic corporeal property of the body in action. The person is predisposed as an enactive mindful body to sense the affinities that pervade his/her existence as part of a wider ecology. This embodied ecological-ness is an important means of dealing with the human as a sensing subject, because Mason (pp. 48–49) distinguishes between embodiment scholarships that relocate subjectivity into a person’s body, and those which extend it through extra-bodily ecologies (e.g. the radical enactivist tradition of cognitive science (Hutto & Myin, 2012)). She prefers the latter, but still, the human, and the human body, remains frontstage in a manner that echoes contemporary SPL attentiveness to the body (Holmes, 2019). Again, here, connective ontology offers a means of rethinking the existential nature of a subject that is essential to SPL, but is often left ontologically indeterminate.

Throughout, there is an implicit humanist hue to connective ontology that sits uneasily beside other post-humanist new-materialisms. Mason criticises strong post-humanism for a well-intentioned but misguided stripping away of human creative energies that renders the world ‘somewhat characterless’ (p. 171). Hence, I would characterise connective ontology as pragmatically humanism-sceptical – content with (even gladly embracing) the frictions and inconsistencies. This position can be sociologically helpful, because it robustly critiques humanism and offers an alternative, without rejecting human vitality altogether and thus undermining empirical efforts to understand social life. Of course, beyond SPL, much sociology is less indebted to the human subject. Hence, we might wonder whether there are entirely non-human affinities (recall the trout). Furthermore, we might even reflect on potential affinities between those things that we would traditionally consider inanimate. Are the relations between table and chair, which intuitively exist in some sense, affinities?

Beginning with non-human life, *Affinities* explicitly opposes humanism regarding questions of ecology and the more-than-human. Mason asks that we ‘pay attention to the meld or the goings on not only of things and human lives, but of other things and other livings’ (p. 124), which extends to ‘technologies’, ‘the climate’ and ‘the sway of life itself’ (p. 125). This indicates that the connections of connective ontology can flow in isolation from human sensibilities. Intuitively, the flowing of more-than-human affinities seems feasible. My rabbit will binky, cluck and flop in joy on particular evenings when the socio-atmospherics are conducive to it, in an ineffable manner that I can sense in the moment, but find difficult to pinpoint as having any particular quality beyond a certain ambiance and energetic charge. I find it plausible that the affinities of connective ontology flow through the rabbit’s life independently of me or any other person.

Regarding inanimate objects, the importance of living and its animating energies can be read as undermining the capacity for objects to be entwined in affinities that do not immediately relate to some form of life (accepting that there is no distinct boundary between the living and non-living [Lane, 2015]). However, while living animation is paramount, non-living things are not without affinities. Writing on possessions, Mason rejects traditional materialism and instead advocates an approach to things in terms of affinities as ‘relational forces and energies that animate and entwine them’ (p. 124). Hence, all things can be said to have lives in a certain respect, or to at least have possibilities of aliveness. In practice, an ecological sensibility makes it difficult to imagine objects that are entirely isolated from affinities. Indeed, Mason questions ‘conventional assumptions about places as material territories, things and technologies as inert objects’ (p. 168). She (p. 50) also disputes the conceptualisation of material agency in terms of affective capacities relative to social action that is ontologically implicit in much SPL research into material objects (Woodward, 2019), and thus could offer a novel grounding for materialist SPL. Hence, pragmatically, I would argue that *entirely inanimate* objects do not exist in (or, at least, are inconsequent to) connective ontology. For instance, while I can imagine an inanimate object in abstract, in practice it would be impossible for me to convey such an object to you without imputing some vitality to said object through my forms of expression.

## Vital animations

The issue of inanimate objects, or objects that are animated through their entwinement with living and the connections that make up that entwinement, can be read as providing some indication of the nature, and perhaps origins, of connections more generally. Life (or rather living in the active, perpetually happening, sense) is at the heart of *Affinities*. This is first and foremost a sociology of personal *life*. Mason (p. 10) argues that through the machinations of its being lived, life animates affinities, as we are living and experiencing those affinities. I contend that this entails two things. First, connective ontology is a social ontology – or perhaps a ‘living ontology’ is a better means of capturing (almost) more-than-human intra-action. It does not *really* speak to ontological concerns beyond (human) life as it is lived, in a manner that agential-realism might, because the living of life underpins affinities’ vital energies. It is not that it absolutely cannot be extended beyond the study of human life, but rather that this is simply not the purpose to which it is tailored (i.e. to do SPL).

Second, connective ontology is instructively resonant with vitalism – the idea that lifeforce transcends conventionally conceived physical processes. Indeed, one could interpret the connection focus of connective ontology as a sensitising method for pinpointing a pragmatic route into studying vitality itself. Exemplifying this vitalism, Mason casts life as a connecting force transcending objects and contexts: ‘Experience is vital – because life is *lived.* . . . There are forces and flows and “worldings” that are not reducible to, for example, bodies, or assemblages . . .: the ontological point being that this is what life is, and not how life relates to a context’ (p. 183). Hence, while connections are the basic unit of connective ontology, they are not inevitable. Affinities are animated by the vitality of lives being lived (p. 2), so even the basic unit is vitally contingent.

Crudely put, vitalism suggests that life entails a force that is distinct from the chemical and physical mechanics traditionally mapped out by the basic sciences. There are distinctive subcategories of vitalism – animist, naturalist, polemicist – but all loosely conceptualise an enlivening energy that transcends isolatable molecular processes. Historically, debates regarding the existence and/or nature of this force animated (forgive the pun) 18th and 19th century biology, and have since been recurrently rejected by the life sciences. Ironically, the recurrency of those rejections has sustained vitalism as a prescient scientific and philosophical concern, a tendency that Canguilhem depicted as the ‘vitality of vitalism’ (Greco, 2005).

Sociologically, vitalism has historically been most influential in the metaphysical work of Simmel, particularly *The View of Life* (1918/2010). Here, vitalism renders (social) life something remarkably similar to connective ontology, a relational flux in a monist universe. Following a neo-vitalist revival in the early 2000s (Lash, 2005), vitalism is now once more a distinct sociological concern (Greco, 2021). Greco (2021, p. 49) notes: ‘There is now explicit talk of a “vitalist turn”, one that would supersede the “discursive” while encompassing the “affective” and the “ontological” (turns).’ This is intriguing because the ontological turn, characterised by a deconstruction of living/non-living duality, has nullified vitalism’s core concern – life – and instead seeks to apply it to matter(ing) wholesale (Osborne, 2016). The result is a *processual vitalism*, less constrained by essentialising a notion of life (as set in contrast to non-life) and more attuned to the ontological turn with its monist rejection of dualities (Greco, 2021).

An appreciation of connective ontology as vitalism-adjacent (in the expansive processual manner critiqued by Osborne) presents an opportunity to push further ontologically, by attending to the energy that comprises the connections at the heart of connective ontology. In *Affinities*, this energy seems to originate from a processual vitalist notion of life, living and liveliness as a foundational force. Going further, we might claim that the basic ontological units of connective ontology are connections(/affinities): those connections are energy, and that energy is vitality or lifeforce. Going further still, I would argue that Mason offers clues regarding the nature of vital connective energy when repeating the idea of ‘emanation’ (p. 45). To my reading, her use of emanation is highly attuned with the concept of dynamic emergence, and it is telling that the importance of the ‘dynamic’ is also repeated throughout *Affinities* (p. 46).

Mason (p. 201) argues that sociology needs to be attuned to the dynamics of ecology as it is lived through connections. Both the dynamic (i.e. perpetual flux) and the dynamic (i.e. emergent properties, greater than the sum of their parts and unattributable to any particular part) are essential to the connective ontology that I trace through *Affinities*. The latter notion of dynamic emergence offers a means of accounting for vital energies. It is exemplified by the tendency for electrically induced fields to develop across complex systems, e.g. computers and brains, and extend out beyond them so that they are detectable from some distance. In both instances, observable fields emerge under their own volition from the overall functioning of the systems, but are not reducible to any singular component or process. These fields have particular rhythmic flows, the organisation of which is also not attributable to any particular thing, and they are generative in and of themselves (Godfrey-Smith, 2020). The vital animating energy that comprises affinities can be similarly envisaged as an emergent, or emanating, property of life as it is lived.

Mason writes that ‘things’ are ‘more than “themselves” as conventionally defined’ (p. 169), and I argue that the energies of affinities can be similarly read as dynamically emergent. Here, a naturalist reader who is sympathetic to traditional materialism could reasonably go further to note that lifeforce is an emergent property of biochemistry, and therefore contend that connective ontology can converge with, and be positioned as emerging from, a conventional ontology of classical physics. Similarly, an animist reader can root connective ontology in a teleological conceptualisation, understanding energy in relation to its effects. Such philosophical debate is beyond the scope of this paper, but gives pause for reflection in the wider context of new-materialist scholarship and the reinvigoration of ontological sensibilities across social science (Fox & Alldred, 2018; Ingold, 2012; Pickering, 2017).

Rather than a straightforward contribution to the grand philosophical issues, such as vitalism and dynamism, amidst which connective ontology might be read, I think it is more fruitful, and in line with Mason’s writing, to consider its stylistic implications. In particular, I reiterate the extent to which connective ontology has been almost entirely unremarked upon in explicit terms throughout its history, and yet has been implemented so sophisticatedly across *Affinities*. It is a revolutionary ontology manifest with intriguing subtlety, nurturing an unassuming radicalness that distinguishes it from many ontological turn sociologies. Stylistically, connective ontology resonates with the world to which it attends, that is, the tantalising magic of the mundanely familiar. This is the ultimate playful creativity of *Affinities* and its implicit treatment of connective ontology. The essential ineffability of social life is captured and communicated ineffably, conjuring a sophisticated form of sociological disposition in the reader.

## Conclusion: Parched connections

This paper began with the observation that SPL has been largely immune to the ontological turn that has enlivened sociology more widely since the mid-2000s (Roseneil & Ketokivi, 2016). In response, some have tried to retrofit ideas that have been popularised elsewhere, e.g. new-materialism, into SPL (Mauthner, 2021). In contrast, my argument is that SPL is fertile (at least implicitly) with its own ontologies, that can be traced, reconfigured and developed by engaging with wider disciplinary developments. This can offer provocations to other sociologies, while retaining useful resonances with existing scholarship. The effort to realise and advocate different ontologies from distinct and unorthodox standpoints is well-aligned with the ethical commitments of the ontological turn generally and parallel calls for ‘live methods’ (Back & Puwar, 2012).

Connective ontology is suited to these ends in various ways. Besides offering a robust and stimulating ontological footing that is often underdefined in SPL, connective ontology simultaneously links SPL into wider sociological concerns from which it can draw inspiration and to which it can contribute insight. In this paper, I have attended to intersections with Barad’s agential-realism, Latourian assemblages and Simmel’s social vitalism, but the possibilities extend further. That latter relation to vitalism is underdeveloped in SPL (again, at least explicitly), but it could prove poignant given recent discussion of a vitalist turn in sociology. An SPL with clearly demarcated vitalist sensibilities could offer a unique focus on the quotidian vitalities of personal life, opening up new research sites and methods to vitalist scholarships.

This extends to the biosocial sociological tradition, wherein classical distinctions between bio and socio stuff are being troubled in relation to everyday practices of doing biology and sociology together (Fitzgerald et al., 2016; Fletcher & Birk, 2020; Meloni et al., 2016). My own empirical work in this domain is exploring connective ontology as a provocation to reimagine environmental effects on cognition. I am using creative methods, inspired by SPL, to explore the sensory experiences of people with dementia during everyday travel. In doing so, I am evaluating post-cognitivist scientific hypotheses regarding emanating cognition that seem profoundly resonant with connective ontology. Indeed, discovering those resonances led me to wonder what others might be out there, as yet not explicated, eventually leading me to write this paper. Again, connective ontology presents possibilities for cultivating unorthodox sociological, and indeed social scientific, connections. Through its marked resonances with the ontological, vitalist and biosocial turns, connective ontology can speak to the nature and scope of contemporary sociology.

In this paper, I have sought to trace that connective ontology as a conception of vitally animated connection units, albeit with remaining (fruitful) tensions regarding the semi-human-centredness of sensations. While I have attended closely to *Affinities* as a guide, the resulting outline offers my interpretation in relation to other literatures. It is not exclusive. *Affinities* is intentionally provocative to stimulate dialogue with readers, and this paper is my own such dialogue. Scholars interested in connective ontology should read *Affinities*, regardless of the observation that connective ontology is never mentioned outright. The ineffability of affinities renders the expansive but implicit realisation of connective ontology in *Affinities* an effective sociological incubator, nurturing a connective disposition in the reader. It guides the reader toward an affinity with affinities, offering a seemingly incidental form of instruction, though of course there is nothing incidental about it.

The opposite of Mason’s artful indication of connective ontology is my rather mechanistic attempt to define it. Through such methodological dissimilarity, I position this paper as something like another facet, refracting new light on the problem and in doing so somewhat changing the problem. This paper provides a different type of treatment of connective ontology that I hope will make it more appealing to some sensibilities, and by extension more used, even if that treatment conceals at the same time that it reveals. Methodologically, this raises questions about how sociologists might best communicate connective sociologies of the ineffable. Mason and I have discussed this in personal communications and we disagree on the relative positions, promises and perils of implicit evocations versus explicit specifications of ontology.

It is important to note that an understated and indirect approach to the ineffable can feel troublingly apolitical. Mason’s focus on the magically mundane can easily be read in ways that are politically agnostic. This may suit some people’s predispositions, but for many will feel at least disappointing, if not actively frustrating, or even deleterious to the entire project. Similarly, new-materialist and post-humanist scholarship has repeatedly been criticised for being naïvely apolitical (Fox & Alldred, 2016). In response, scholars have worked to develop more critically engaged approaches that furnish workable foundations for transformative action (e.g. Fox & Alldred, 2018, 2022; Giraud, 2019). In the case of connective ontology, one remedy could be to nurture an attentiveness to the sociopolitics of connection, an approach proposed by Bonelli and Vicherat Mattar (2017) in relation to indigenous political struggle in Chile. Variations of connective ontology can likely be turned to a vast array of sociopolitical issues.

As an example, I return to the humanism-sceptical connective ontology of trout and rabbit lives, and the potential for a more politically engaged connective sociology of ecology. I am writing this in a garden rendered almost entirely brown by an unprecedented heatwave, while nursing lungs that are suffering the aftereffects of a second bout of Covid. I am, momentarily, highly sensitised to the inevitability of ecological holism, an inevitability recognised and emphasised by connective ontology. The dead saplings, my heavy chest, the fish gasping for air in its diminishing pond, all constitute foreboding (dis)connections in need of profound reconfiguration. The affectiveness of these connections is enlivening new connections in response. I have never appreciated the air that I breathe or the grass that I walk on as much as I do now, nor have I ever been so inclined to animate new connections.
